# Supplementary material for: Oligomerization of the FliF Domains Suggests a Coordinated Assembly of the Bacterial Flagellum MS Ring
Source: Front Microbiol. 2022 Jan 11;12:781960. doi: 10.3389/fmicb.2021.781960 (PMC8786727; doi:10.3389/fmicb.2021.781960)
Supplement: Supplementary file 1 [file Table_1.docx]

| Domain | Construct boundaries | Monomer MW (KDa) | Oligomeric state of the purified protein | Elution Volume (Ve, mL) | Apparent molecular weight (KDa) | Expected molecular weight (KDa) |
| --- | --- | --- | --- | --- | --- | --- |
|  |  |  |  |  |  |  |
| RBM1+L1 | 50-124 | 8.13 | Monomer | 18.90 | 7.56 | 8.13 |
| RBM2+L2 | 124-229 | 11.21 | Oligomer | 9.35 | 390.15 | 257.83 |
| RBM1+L1+RBM2+L2 | 50-229 | 19.25 | Monomer | 17.65 | 11.93 | 19.25 |
| RBM3 | 231-438 | 22.50 | Oligomer | 8.66 | 868.80 | 765.00 |
| RBM1+RBM2+RBM3 | 50-438 | 41.87 | Oligomer | 8.27 | 1046.44 | 1422.56 |
| Full length | 1-560 | 63.95 | - | - | - | - |
